# The developmental dynamics of the *Populus* stem transcriptome

**DOI:** 10.1111/pbi.12958

**Published:** 2018-06-25

**Authors:** Qing Chao, Zhi‐Fang Gao, Dong Zhang, Biligen‐Gaowa Zhao, Feng‐Qin Dong, Chun‐Xiang Fu, Li‐Jun Liu, Bai‐Chen Wang

**Affiliations:** ^1^ Key Laboratory of Photobiology Photosynthesis Research Center Institute of Botany Chinese Academy of Sciences Beijing China; ^2^ University of Chinese Academy of Sciences Beijing China; ^3^ Biomarker Technologies Corporation Beijing China; ^4^ The Key Laboratory of Plant Molecular Physiology Institute of Botany Chinese Academy of Sciences Beijing China; ^5^ Key Laboratory of Biofuels Qingdao Engineering Research Center of Biomass Resources and Environment Qingdao Institute of Bioenergy and Bioprocess Technology Chinese Academy of Sciences Qingdao Shandong China; ^6^ College of Forestry Shandong Agricultural University Tai‐An Shandong China

**Keywords:** PacBio Iso‐Seq, stem cell maintenance, primary growth, secondary growth, transcription factor

## Abstract

The *Populus* shoot undergoes primary growth (longitudinal growth) followed by secondary growth (radial growth), which produces biomass that is an important source of energy worldwide. We adopted joint PacBio Iso‐Seq and RNA‐seq analysis to identify differentially expressed transcripts along a developmental gradient from the shoot apex to the fifth internode of *Populus* Nanlin895. We obtained 87 150 full‐length transcripts, including 2081 new isoforms and 62 058 new alternatively spliced isoforms, most of which were produced by intron retention, that were used to update the *Populus* annotation. Among these novel isoforms, there are 1187 long non‐coding RNAs and 356 fusion genes. Using this annotation, we found 15 838 differentially expressed transcripts along the shoot developmental gradient, of which 1216 were transcription factors (TFs). Only a few of these genes were reported previously. The differential expression of these TFs suggests that they may play important roles in primary and secondary growth. AP2, ARF, YABBY and GRF TFs are highly expressed in the apex, whereas NAC, bZIP, PLATZ and HSF TFs are likely to be important for secondary growth. Overall, our findings provide evidence that long‐read sequencing can complement short‐read sequencing for cataloguing and quantifying eukaryotic transcripts and increase our understanding of the vital and dynamic process of shoot development.

## Introduction

All woody plants and some non‐woody trees undergo primary and secondary growth. During primary growth, the primary meristems, including the shoot apical meristem (SAM) and procambium, which were already established during embryogenesis, maintain apical growth by providing stem cells to the primary growth zone (Aichinger *et al*., 2012; Miyashima *et al*., 2013; Ohashi‐Ito and Fukuda, 2010; Weigel and Jurgens, 2002). Once the primary vascular tissues that are derived from procambium are established, the procambium develops into the cambium, which produces cells that develop into vascular tissues. This stage indicates the beginning of secondary growth where the shoot begins to undergo radial growth that results in increased girth. In trees, the biomass produced by secondary growth is a source of biofuel, and understanding how the transition from primary to secondary growth is regulated can aid efforts to improve biomass production.

Several genes involved in the regulation of primary growth from the SAM, which provides cells for apical growth and lateral organogenesis, have been identified. The SAM consists of a central zone (CZ), a surrounding peripheral zone (PZ) and an underlying rib zone (Aichinger *et al*., 2012). In the CZ, pluripotent stem cells and the organizing centre (OC) together form a stem cell niche. In *Arabidopsis thaliana*, a regulatory loop between the *CLAVATA* (*CLV*) and *WUSCHEL* (*WUS*) genes maintains stem cell fate in the SAM (Schoof *et al*., 2000). *SHOOT MERISTEMLESS* (*STM*) also competes with *CLV* to initiate and maintain the SAM in *Arabidopsis* (Clark *et al*., 1996). The *CUP‐SHAPED COTYLEDON* (*CUC*)/*STM* regulatory pathway is associated with the establishment of organ boundaries and the initiation of the SAM (Aida *et al*., 1999; Long and Barton, 1998).

Different sets of genes regulate lateral organ development from the PZ, which is located at the tip of the meristem. In these regions, small groups of cells show signs of differention based on upregulation of markers for asymmetric cell division and differentiation in *Arabidopsis*,* ZWILLE/PINHEAD* (*ZWI*), *PIN‐FORMED1* (*PIN1*), and *REVOLUTA* (*REV*) (Aida *et al*., 2002; Carraro *et al*., 2006; Lynn *et al*., 1999; Otsuga *et al*., 2001). *AINTEGUMENTA* (*ANT*) and *LEAFY* (*LFY*) are subsequently activated, but in a symmetric manner.

The following section of primary growth along the longitudinal direction is the procambium. The procambium asymmetrically gives rise to xylem and phloem precursor cells, which ultimately differentiate into the centripetal xylem and centrifugal phloem to form vascular bundles that are arranged along a ring passing through the ground tissue (Baucher *et al*., 2007; Carlsbecker and Helariutta, 2005). In *Arabidopsis* embryos and leaves, the polar auxin efflux protein PIN1 is activated by the HD‐ZIPIII protein *Arabidopsis thaliana* Homeobox Gene 8 (ATHB8), which may in turn be activated by MONOPTEROS (MP)/AUXIN RESPONSE FACTOR5 (ARF5) (Aida *et al*., 2002; Donner *et al*., 2009; Ohashi‐Ito and Fukuda, 2010). These processes form a positive regulatory loop from auxin to MP‐ATHB8‐PIN1, and PIN1 also participates in auxin flow (Miyashima *et al*., 2013). But the regulatory mechanism in poplar is unclear.

Secondary growth takes place when the vascular cambium initials differentiate from the procambium within the vascular bundles (fascicular cambium) and from parenchymatous rays in the interfascicular regions (interfascicular cambium) (Baucher *et al*., 2007). Similar to primary growth, most of the genes involved in secondary growth have been identified based on molecular genetic studies in *Arabidopsis*, which has a well‐annotated genome sequence available. But research has mainly focused on the *Arabidopsis* root. In *Arabidopsis*,* DOF5.6/HCA2* contributes to the regulation of interfascicular cambium formation and vascular tissue development (Guo *et al*., 2009). The MYB transcription factor (TF) *ALTERED PHLOEM DEVELOPMENT* (*APL*) is required for phloem differentiation and inhibition of xylem differentiation in the phloem positions in *Arabidopsis* roots (Bonke *et al*., 2003). The NAC domain TFs, SECONDARY WALL‐ASSOCIATED NAC DOMAIN PROTEIN1 (*SND1*), VASCULAR‐RELATED NAC‐DOMAIN6 (*VND6*) and *VND7*, play essential roles in xylem vessel differentiation in *Arabidopsis* (Kubo *et al*., 2005; Zhong *et al*., 2006). Each activates the expression of 12 downstream TF genes, which are mostly MYBs, or directly affects the transcription of genes associated with lignin, cellulose and hemicellulose biosynthesis (Wang and Dixon, 2012). The feedback mechanism between PXY‐CLE41 can affect vascular cell division and determine the orientation of cell division in aspen and *Arabidopsis* (Etchells and Turner, 2010; Fisher and Turner, 2007).

Some of the pathways that regulate secondary growth in *Arabidopsis* are conserved in trees. For example, PXY‐CLE41 signalling acts to regulate both the rate of cambial cell division and secondary growth in aspen (*Populus tremuloides*) (Etchells *et al*., 2015). In *Populus*, the same type of regulatory mechanism involving the *ARBORKNOX1* (*ARK1*, ortholog of *STM*) genes has been shown to control cell fate in the vascular cambium (Du *et al*., 2009; Groover *et al*., 2006; Liu *et al*., 2015). *PtaLBD1* promotes the expression of *APL* and restricts the expression of *ARK1* and *ARK2*, which are involved in maintaining the cambium meristem (Yordanov *et al*., 2010).

*Populus* is used as a model system for secondary growth because it has better wood formation than the model plant *Arabidopsis* and is an important source of biofuel (Jansson and Douglas, 2007). However, secondary growth in the *Populus* shoot is quite different from that in the *Arabidopsis* root and it is therefore necessary to identify the genes that regulate secondary growth in poplar (Du and Groover, 2010). Since the release of the black cottonwood tree, *Populus trichocarpa*, draft genome (Tuskan *et al*., 2006), more genes have been discovered. But not all the functional transcripts have been identified. Microarray and RNA‐seq technologies have also been extensively used to analyze *Populus* cambium development, vascular tissue formation and phloem and xylem differentiation during secondary growth (Aspeborg *et al*., 2005; Hertzberg *et al*., 2001; Ko *et al*., 2012a; Schrader *et al*., 2004; Sundell *et al*., 2017). In one study, 271 differentially expressed fragments that represent genes that may be involved in the regulation of secondary growth were sequenced from different stem segments (Prassinos *et al*., 2005). In a microarray study, 3016 genes were found to be differentially expressed during stem development (Dharmawardhana *et al*., 2010). But due to technical limitations, these transcripts represent far less than the 28 294 annotated genes that have been found to be expressed during cambial growth and wood formation by RNA‐seq methods (Sundell *et al*., 2017).

Short‐read sequencing using the Illumina platform, is a powerful method for quantifying gene expression. However, the limitations of short‐read sequencing lead to a number of computational challenges and hamper transcript reconstruction and the detection of splice events. In the last few years, an increasing number of Pac‐Bio full‐length transcriptomes have been generated. The PacBio Iso‐Seq (isoform sequencing) platform provides long reads—often up to 10 kb, making it possible to accurately reconstruct full‐length splice variants. These studies have led to the discovery of thousands of novel genes and alternatively spliced isoforms in several species, including human, sorghum, maize, yeast, moso bamboo and cotton (Abdel‐Ghany *et al*., 2016; Wang *et al*., 2016, 2017, 2018). Also, third‐generation sequencing of human embryonic stem cells combined with previously generated EST and RNA‐seq databases uncovered 2103 novel isoforms, ~10% of which were derived from unannotated genetic loci, indicating that full‐length isoform identification and quantification can generate a high‐confidence isoform dataset (Au *et al*., 2013). Overall, the above studies provide evidence that long‐read sequencing complements short‐read sequencing in cataloguing and quantifying eukaryotic transcripts and can aid in finding more alternatively spliced isoforms.

Here, we adopted joint PacBio full‐length sequencing and RNA‐seq analysis to identify specific genes and pathways involved in the transition from primary to secondary growth in *Populus*. We obtained 15 838 differentially expressed transcripts (DETs) from the apex and different sections of the stem, including 1216 TFs. The differentially expressed transcripts may be related to primary and secondary growth.

## Results and discussion

### Anatomic analysis of the *Populus* stem

**Figure 1:**
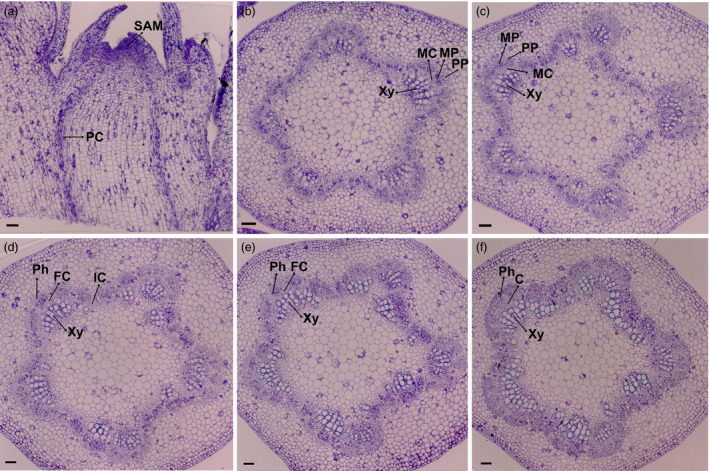
Anatomic analysis of the poplar apex and stem. (a) Cross section of the shoot apex. (b) Transverse section of internode 1. (c) Transverse section of internode 2. (d) Transverse section of internode 3. (e) Transverse section of internode 4. (f) Transverse section of internode 5. SAM: shoot apical meristem, PC: procambium, MC: metacambium, PP: protophloem, MP: metaphloem, Xy: xylem, Ph: phloem, FC: fascicular cambium, IC: interfascular, C: cambium. Scale bars, 50 μm.

To determine key factors that control the transition from primary growth to secondary growth, we used PacBio to sequence the transcriptome of *P. deltoides* × *P. euramericana* cv. ‘*Nanlin895*’ stems undergoing these transitions. Because it is widely planted in south China, grows quickly and is easily transformed (Song *et al*., 2011; Zhao *et al*., 2013; Zhu *et al*., 2013), we used the ‘*Nanlin895*’ cultivar for this study. To determine which plant materials to combine for PacBio sequencing, we performed anatomical analysis of the shoot apex (hereafter referred to as apex) and successive internodes below the apex, referred to as plastochron indices IN1, IN2, IN3, IN4 and IN5, respectively, as described in a previous study (Larson and Isebrands, 1971). The apex consists of an apical dome zone of stem cells and a peripheral zone where leaf primordia initiate. The cells located in the SAM have a relatively flat shape (Figure 1). And the procambium, which gives rise to primary phloem and xylem, initiates acropetally through the residual meristem beneath the SAM. The primary phloem and xylem are dominant in IN1, IN2 and IN3, as described previously (Dharmawardhana *et al*., 2010). The first signs of interfascicular parenchyma cells that differentiate into a full ring of cambium initials are visible in IN3, indicating the beginning of radial secondary stem growth. IN5 has well‐developed secondary phloem tissue and secondary xylem vessels, as well as fibres with well‐lignified secondary walls (Figure 1). With the development of the vascular cambium and initiation of secondary growth in IN4‐5, stem elongation ceases while the diameter of the stem girth continues to increase. From these observations, IN4‐5 represents secondary growth. Therefore, we selected these internodes to represent the transition from primary growth to secondary growth.

### Sequencing of the *Populus* transcriptome using the PacBio Iso‐Seq platform

To identify genes that regulate the SAM and the transition from primary to secondary growth in ‘*Nanlin 895*’, we sequenced the transcriptomes of different regions of the *Populus* stem using the PacBio Iso‐Seq (isoform sequencing) platform. This platform provides long reads of complete isoforms. To identify transcripts that are as long as possible, high‐quality RNA was used for Iso‐Seq (see Methods for details). Total RNA was extracted from three regions of the *Populus Nanlin895* stem: (i) apex, (ii) a mixture of IN1‐3, and (iii) a mixture of IN4‐5. To avoid loading bias, which is the preferential sequencing of shorter transcripts, multiple size‐fractionated libraries including 0–1, 1–2, 2–3 and 3–10 kb libraries were made using a SageELF device. Libraries were sequenced on the PacBio RS II platform using the latest P6–C4 chemistry with 27 SMRT cells, yielding 1 613 676 Reads of Inserts (ROI), of which 887 877 (55%) were full‐length (containing the 5′ barcoded primer, 3′ barcoded primer and the poly (A) tail) (Data S1).

**Figure 2:**
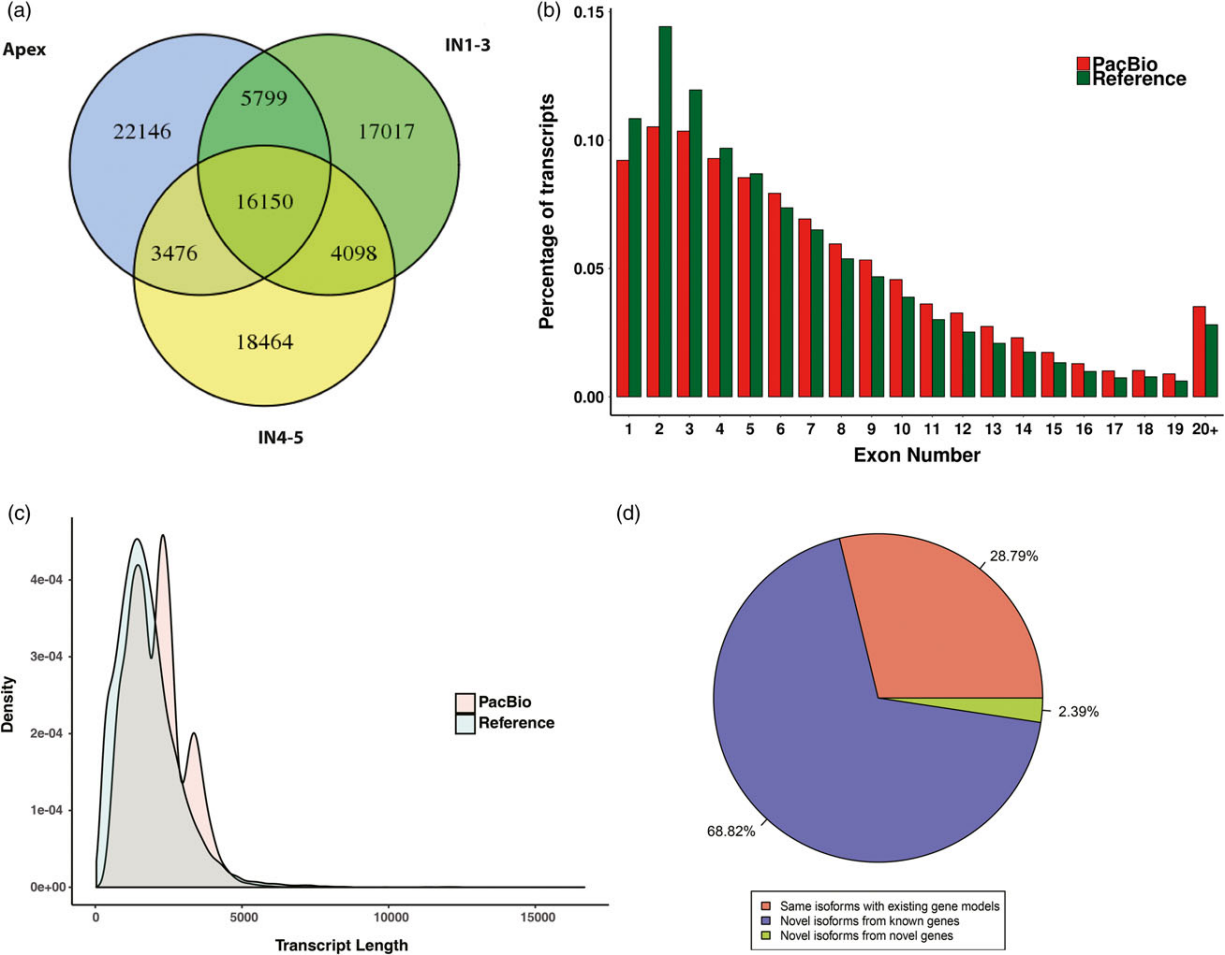
Comparison of *Populus trichocarpa* v3.0 (Reference) and PacBio Iso‐Seq data isoform annotations. (a) Identified isoforms in three samples (Apex, IN1‐3 and IN4‐5). (b) Distribution of the percentage transcripts with different exon numbers for reference and PacBio Iso‐Seq data. (c) Comparison of gene model and PacBio Iso‐seq isoform length. (d) A pie chart showing the percentage of PacBio Iso‐Seq isoforms that are the same as existing gene models, novel isoforms of known genes and novel isoforms of novel genes.

We used an isoform‐level clustering algorithm, Iterative Clustering for Error Correction (ICE) (Gordon *et al*., 2015), to improve consensus accuracy and polished full‐length consensus sequences from ICE using RS_IsoSeq (v2.3.0). We obtained 308 549 consensus isoforms, of which 234 687 were high‐quality consensus transcript sequences. High‐quality transcript sequences were mapped to the *Populus* genome (Phytozome, *Populus trichocarpa* Pt_v3.0) using GMAP. Short‐read Illumina sequencing of the same samples was done to quantify the Iso‐Seq non‐redundant isoforms. ToFu (Gordon *et al*., 2015) processing yielded 87 150 non‐redundant isoforms (Table 1). Of the three tissues, the apex had the highest proportion of tissue‐specific isoforms (22 146; 26.2%), followed by IN4‐5 (18 464; 21.6%), and IN1‐3 (17 017; 19.9%) (Figure 2a). Longer isoforms were identified from Iso‐Seq than from the reference database (Pt_v3.0) and more exons were found in this study (Figure 2b,c). The non‐redundant transcript isoforms were used in subsequent analyses (Figure S1).

**Table 1:** PacBio Iso‐Seq output statistics

Samples	Number of SMRT cells	Polymerase reads	Number of insert reads	Number of insert base reads	Number of five prime reads	Number of three prime reads	Number of poly‐A reads	Number of full‐length reads	Number of full‐length non‐chimeric reads	Average full‐length non‐chimeric read length	Full‐length percentage (FL%)
Apex	9	1 352 628	604 385	1 191 087 341	379 572	407 917	400 207	320 351	315 844	7822	53.00
IN1‐3	9	1 352 628	560 769	1 058 569 542	362 406	387 945	380 417	310 043	306 526	7743	55.29
IN4‐5	9	1 352 628	448 522	853 915 854	304 979	322 000	317 875	268 186	265 507	8221	59.79

### Isoform detection and tissue‐specific alternative spliced isoforms

We compared the 87 150 isoforms against the *Populus* genome gene set (Pt_v3.0), which contains 71 013 isoforms from 41 335 genetic loci. We classified isoforms into three groups as follows: (i) 25 092 known isoforms detected by Iso‐Seq and mapped to the gene set, (ii) 59 977 new isoforms of annotated genes and (iii) 2081 isoforms, which belong to 1575 likely novel genetic loci, that do not overlap with any annotated gene (Figure 2d). In a study where the maize transcriptome was obtained by single‐molecule long‐read sequencing, out of 111 151 isoforms identified, 62 547 (57%) were new isoforms and 2803 encoded 2253 new genes. In a sorghum study, 27 860 isoforms were identified, including 11 342 (40.7%) new isoforms and 3141 isoforms encoding new genes. The high percentage of new isoforms identified by us and other groups demonstrates that PacBio full‐length sequencing can provide a more comprehensive set of isoforms than next‐generation sequencing.

To determine if the 1575 putative novel *Populus* genes are present in other plants, we conducted BLASTX searches against Swiss‐Prot (*E*‐value ≤e−10; see Methods). In total, 965 (46.3%) of these isoforms were annotated in the Swiss‐Prot database, and the remaining isoforms were unannotated. We integrated the 2081 transcripts from new genes and the 59 977 new isoforms of known genes to improve the *Populus* reference genome annotation (Data S2). By combining our newly identified isoforms with the 71 013 existing isoforms in the *Populus* gene set, we generated an updated *Populus* reference sequence database containing 135 071 isoforms of 42 910 genetic loci.

**Figure 3:**
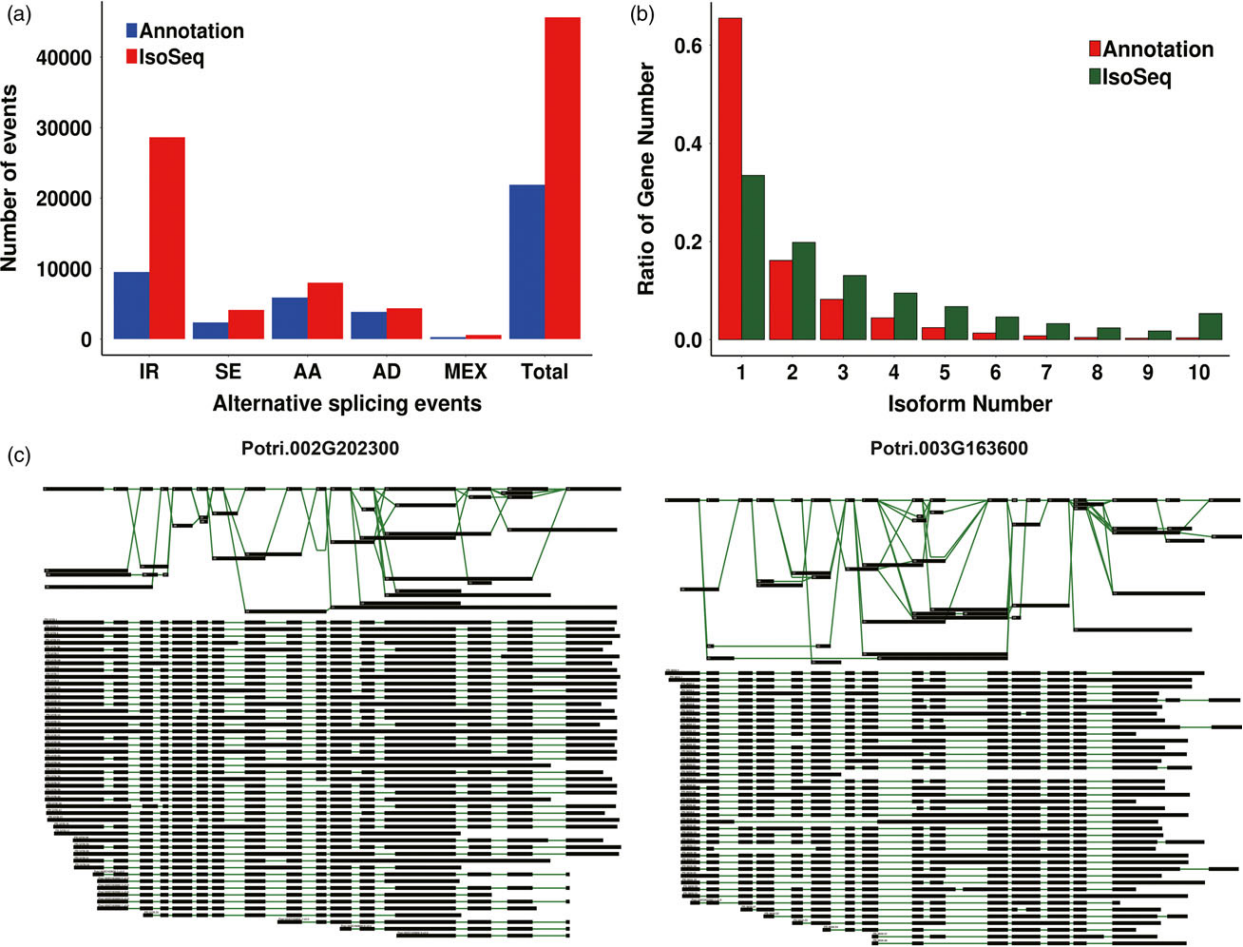
Alternative splicing events and different isoforms from Iso‐seq. (a) The total number of alternative splicing (AS) events in genes based on Iso‐Seq data and annotated gene models. Annotation, AS events in genes based on gene models; Iso‐Seq, AS events in genes based on Iso‐Seq reads. IR, intron retention. SE, exon skipping. AA, alternative 3′ splice site. AD, alternative 5′ splice site. MEX, mutually exclusive exon. (b) The number of genes with one or more splice isoforms based on gene annotation and Iso‐Seq data. (c) Different isoforms of Potri.002G202300 and Potri.003G163600.

We used AStalavista (Foissac and Sammeth, 2007) to determine the number of transcripts in the apex, IN1‐3 and IN4‐5 generated by each of the five main types of alternative splicing (intron retention, exon skipping, alternative 3′ splice site, alternative 5′ splice site and mutually exclusive exon). Among all alternative splicing events, intron retention predominated, accounting for 58.15–64.2% of alternatively spliced transcripts (Figure 3a, Data S3). There were more alternatively spliced transcripts found among Iso‐Seq transcripts compared with previously annotated transcripts (Figure 3b). We found that several genes reported to be important for plant development had more isoforms than previously annotated, such as sucrose synthase *Pt‐SUS2.2* (Potri.002G202300, 23 isoforms) and auxin response factor *PtARF2* (Potri.003G163600, 18 isoforms) (Figure 3c). We selected three genes *ARF8* (PB.6368), *SUTR11* (PB.8036) and *CYP707A4* (PB.13883) and verified the production of the isoforms by RT‐PCR (Figure S2) and found that there was more than one isoform of these genes. Interestingly, different isoforms of *CYP707A4* were expressed during primary and secondary growth.

### Long non‐coding RNA identification

Long non‐coding RNAs (lncRNAs) constitute an important class of regulators of gene expression in most eukaryotes (Flynn and Chang, 2014). We used four computational approaches (see Methods) to identify lncRNAs from the 87 150 PacBio Iso‐Seq isoforms. By filtering and excluding transcripts with an open reading frame (ORF) > 300 bp (Clamp *et al*., 2007), we finally obtained 1187 lncRNAs (493 in the apex, 413 in IN1‐3 and 467 in IN4‐5). Only 33 lncRNAs were found in all three samples (Data S4).

**Figure 4:**
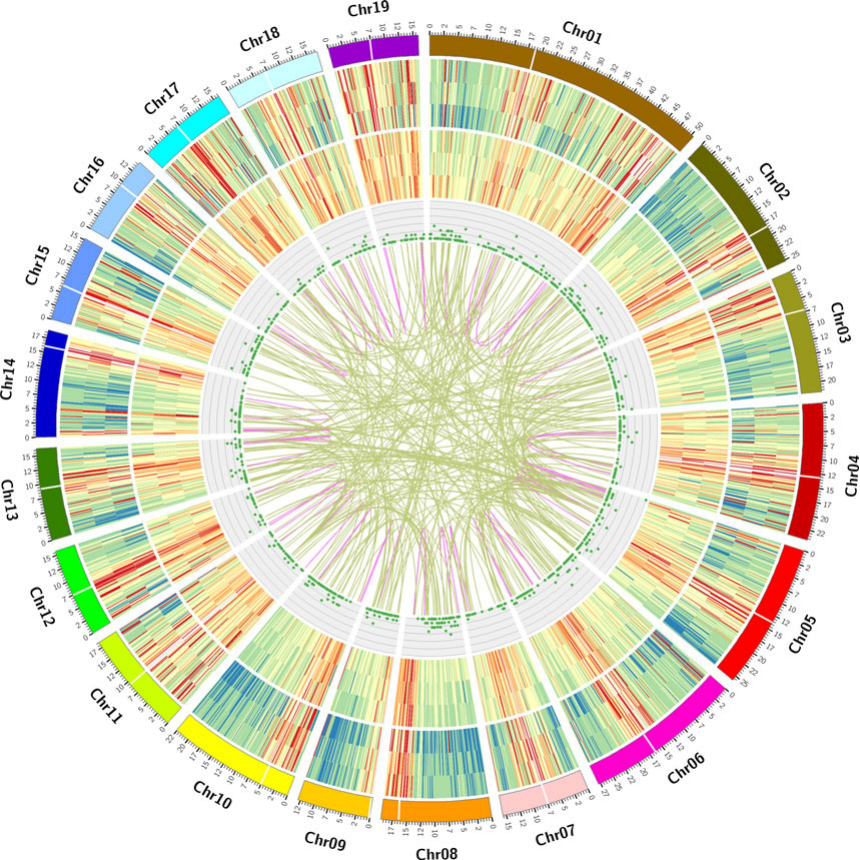
CIRCOS visualization of genomic and transcriptomic features. (a) Poplar chromosomes. (b) Gene density of different samples (Apex, IN1‐3, and IN4‐5). (c) Transcript density of different samples (Apex, IN1‐3, and IN4‐5). (d) Long non‐coding RNA (IncRNA) distribution. (e) Fusion transcript distribution: intra‐chromosome (purple); inter‐chromosome (yellow). The location of centromere regions is labelled according to previous research (Pinosio *et al*., 2016).

The length of lncRNAs varied from 273 to 5560 bp, with the majority (>63%) having a length ≤1000 bp. The mean length was 1052 bp, which was much shorter than the mean length of all 87 150 isoforms (2417 bp). In *Populus*, lncRNAs contain an average of 1.77 exons compared with 7.10 for mRNAs. Mapping lncRNAs to chromosomes revealed that they have a distribution similar to that of mRNAs; both are enriched outside of pericentromeric regions (Figure 4). By comparing the 1187 lncRNAs to *Populu*s lncRNAs (PNRD database), 553 were identified. However, only 31 were previously annotated, which indicates that more transcripts can be identified with PacBio Iso‐Seq than with other methods.

### Fusion transcript identification

A fusion transcript is a chimeric RNA encoded by a single fusion gene or by two different genes that are subsequently joined by trans‐splicing. Generation of fusion transcripts involves the splicing machinery, suggesting either trans‐splicing of distinct genes or splicing of chimeric genes formed by somatic chromosomal rearrangements. Gene fusion, which is caused by somatic chromosomal rearrangement, is a common feature in humans, but only a few examples of gene fusion have been described in plants. Long‐distance movement of a homeobox fusion from mutants to wild‐type scions in grafting experiments causes changes in leaf morphology (Kim *et al*., 2001; Wang *et al*., 2016). Here we identified 356 full‐length transcripts that map to two or more loci in the genome and thus are considered fusion transcripts (Data S5). The highest number of tissue‐specific fusion transcripts was found in the apex (119), followed by IN4‐5 (117) and IN1‐3 (78). Only 14 fusion transcripts were found in all three samples. The fusion transcripts were validated using 18 transcriptome datasets (described below). Specifically, fusion transcripts were required to have at least one uniquely mapping paired‐end read using bowtie 2 (Langmead and Salzberg, 2012), and 25 transcript fusions met this criterion. Gene ontology (GO) analysis of fusion transcripts revealed that most were associated with ATP binding (22 genes), transmembrane transport (10 genes) and proteolysis (5 genes).

### PacBio full‐length sequencing extends the *Populus* annotation and increases the accuracy of transcript quantification

The *P. trichocarpa* genome has been sequenced and annotated, but due to technical limitations, transcript sequences and annotations continue to be updated as more data become available. For example, the latest annotation (Pt_v3) contains more annotations than the previous versions because RNA‐seq data have been integrated (Liu *et al*., 2014). The *P. trichocarpa* genome is also used as a reference for other *Populus* species, but due to species divergence, low mapping ratios are observed when mapping transcriptomes to the current annotation (Chen *et al*., 2015; Liu *et al*., 2014). In order to compare the expression level determined by RNA‐seq based on PacBio reads, eighteen mRNA samples from six different tissues (Apex, IN1‐IN5) were subjected to 2 × 150 bp paired‐end sequencing using the HiSeq platform. In total, 414.2 million trimmed reads were actually mapped to the reference genome, of which 388.8 million reads (88.8%) mapped within known exons in the updated Nanlin895 annotation database (Data S6). In total, we quantified 126 690 isoforms from all samples. The mapped RNA‐seq reads were used to quantify all Pac‐Bio Iso‐seq isoforms from each sample based on the fragments per kilobase of exon per million fragments mapped (FPKM) values. Hierarchical clustering analysis of RNA‐Seq data showed that the data from biological repeats clustered together (Figure S3).

### Different regulatory mechanisms have been adopted for primary and secondary growth

**Figure 5:**
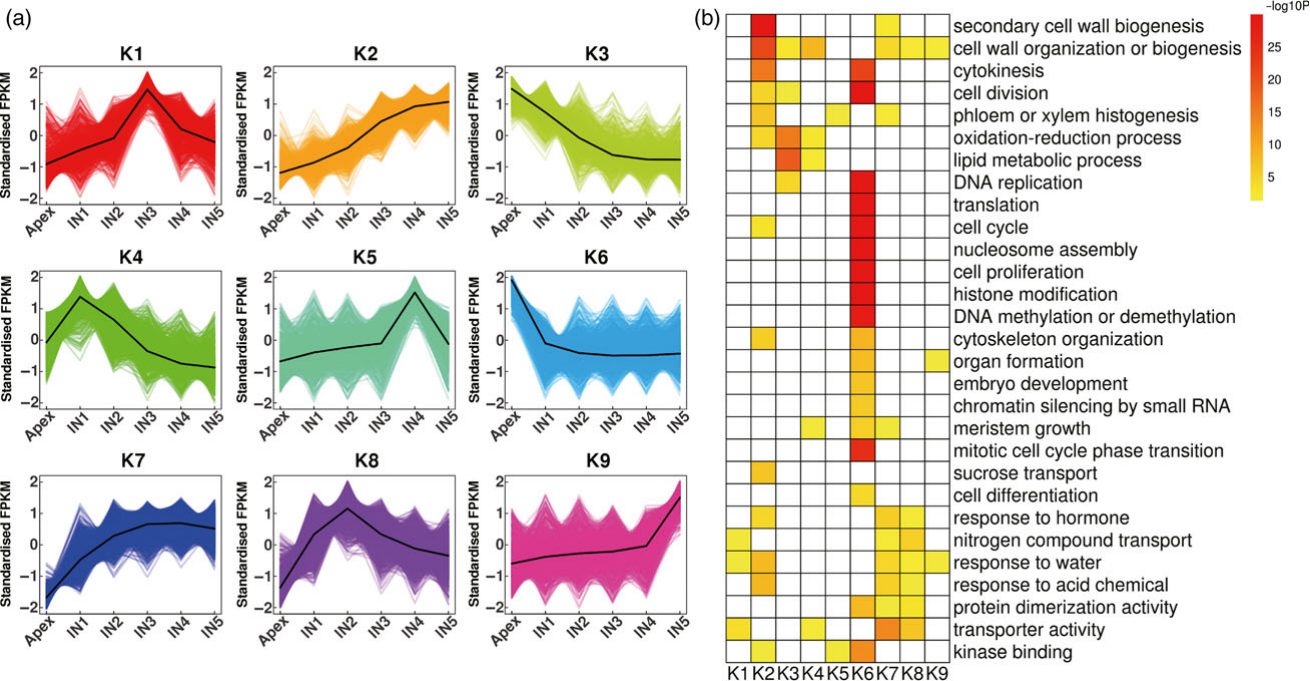
K‐means clustering and enrichment analysis of transcripts differentially expressed between the apex and IN1‐5. (a) K‐means clustering showing the transcriptome expression profiles. Nine clusters were identified based on expression levels in six developmental zones (Apex, IN1, IN2, IN3, IN4 and IN5). (b) Gene Ontology enrichment among the nine clusters. Yellow to red, significant enrichment; white, not significant.

To identify key factors involved in the transition from primary growth to secondary growth, we identified 15 838 DETs from 9950 differentially expressed genes (DEGs) based on RNA‐seq data, which accounted for 18.17% of all isoforms identified in this study (Data S7). Among all transcripts differentially expressed between two adjacent stem regions, most (2747) were identified as being differentially expressed between the apex and IN1, and the smallest number of DETs (183) were identified between IN4 and IN5. Using the K‐means clustering algorithm, 15 834 DETs were grouped into 9 clusters (K1‐9), which represent 99.97% of all DETs (Figure 5a, Data S8). Transcripts from the K6, K4, K8, K1, K5 and K9 clusters were predominantly expressed in the apex and IN1‐5, respectively. Expression levels of transcripts from the K2 cluster increased gradually from the apex to IN5, and the expression level was highest in IN4‐5, which indicates that these transcripts are potentially involved in the transition to secondary growth. Expression levels of transcripts from the K7 cluster increased gradually from the apex to IN3 and reached a steady state in IN4‐5, which was similar to the expression pattern of K2 transcripts. In contrast, the expression levels of transcripts from the K3 cluster decreased from the apex to IN3 and reached a steady state in IN4‐5. These transcripts are potentially involved in primary growth.

To better understand the functions of the genes expressed in different regions of the stem, we performed GO enrichment analysis with the updated annotation as the background (Figure 5b, Data S9). Most enriched GO terms were found for the transcripts differentially expressed during the transition from the apex to IN1 (K6), which is consistent with this transition having the most differentially expressed transcripts. Previous studies have reported that histone modification, DNA methylation or demethylation, and chromatin silencing by small RNAs are important for plant development (Feng *et al*., 2010), and in this study, genes involved in these processes were enriched in the apex. These genes include *Decrease in DNA Methylation 1* (*DDM1*), *DDM2*,* chromomethylase 3* (*CMT3*), *AGO4*,* AGO6*,* ATXR6/SDG34*,* ASHH3/SDG7*,* ASHR3/SDG4* and *CURLY LEAF* (*CLF/SDG1*). DDM1, DDM2 and CMT3 are required to maintain CG and CHG methylation in *Arabidopsis* and rice (Gendrel *et al*., 2002; Habu *et al*., 2015; Jackson *et al*., 2002; Numa *et al*., 2015; Ronemus *et al*., 1996). SDG1 and SDG34 are responsible for methylation of H3K27, which is required for transcriptional repression (Jacob *et al*., 2009; Shafiq *et al*., 2014; Wood *et al*., 2006). ASHH3/SDG7 and ASHR3/SDG4 are members of the trithorax group (TrxG) family, but their function is unknown (Baumbusch *et al*., 2001). AGO4 and AGO6, which were previously reported to be expressed in the SAM of *Arabidopsis* (Havecker *et al*., 2010), may act sequentially to mediate RNA‐directed DNA methylation (Duan *et al*., 2015). High expression of these epigenetic‐related genes in the *Populus* apex, suggests that they may control the expression of a large number of different genes in the SAM.

Transcripts that were predominantly expressed in the apex (K3 and K6) and IN4‐5 (K2) function in similar pathways, such as DNA replication, cell cycle, cell division, cytokinesis and cytoskeleton organization, but genes from different families of cyclin and cyclin‐dependent kinases, E2Fs, and different members of the microtubule‐associated protein (MAP) family were found in each cluster. Expression levels of cyclin A, B, D and cyclin‐dependent kinases A and B, *E2F3*,* E2F TARGET GENE 1* (*ETG1*) and *DP‐E2F‐LIKE 1* (*DEL1*) were highest in the apex. The genes that were highly expressed in the apex are canonical cell cycle genes, except for *DEL1*, which was previously reported to be a repressor of *E2FA* and to be regulated by light in *Arabidopsis* (Berckmans *et al*., 2011; Inzé and Veylder, 2006). Peak expression of P‐type cyclins (CYCPs) was observed in IN4‐5. *CYCP2;1* was reported to promote meristem cell division mediated by *STIP*/*WOX9* and sucrose signals in *Arabidopsis* (Peng *et al*., 2014). In our study, the expression patterns of CYCPs suggest that they may mainly regulate secondary growth. Different MAPs can affect the organization and functions of microtubule arrays, which can control the direction of cell division and expansion during plant morphogenesis and development (Hamada, 2014). For example, *MAP70‐5* was found to be upregulated upon xylem tracheary element differentiation and to regulate secondary wall patterning in *Arabidopsis* wood cells (Pesquet *et al*., 2010). Consistent with different functions of *MAPs* during primary and secondary growth, we found that *MAP65‐6*,* MAP65‐8* and *MAP70‐5* were expressed primarily in IN4‐5, but *MAP65‐1*,* MAP65‐3*,* MAP65‐5* and *MAP70‐1* were expressed primarily in the apex.

The expression patterns of cell cycle‐ and cell division‐associated DETs may give insight into their roles in the regulation of cell division during primary and secondary growth. The SAM can be divided into distinct cell layers; cells from the epidermal layer L1 and subepidermal layer L2 undergo anticlinal cell divisions, while cells from the corpus or L3 layer divide in many planes (Meyerowitz, 1997). In contrast, during secondary growth, the vascular cambium increases the stem diameter and circumference by undergoing periclinal divisions and anticlinal divisions, respectively, resulting in a developmental continuum of secondary phloem and xylem (Chaffey, 1999). Thus, transcripts differentially expressed during primary and secondary growth may play different roles in anticlinal and periclinal divisions.

### Primary and secondary cell wall‐associated transcripts are predominantly expressed in different regions of the stem

During *Populus* shoot growth, primary cell walls play a vital role in the expansion of actively growing cells, and secondary cell walls play a larger role in water transport and mechanical support. Consistent with the different roles of primary and secondary cell walls, GO enrichment analysis indicated that expression of genes involved in cell wall organization or biogenesis peaked in IN1 (K4) and IN4‐5 (K2). Cell wall modification and pectin catabolic process genes were expressed in IN1, and secondary cell wall biogenesis genes were expressed in IN4‐5, which was also enriched in genes involved in phloem or xylem histogenesis.

In total, the expression levels of 469 protein‐coding transcripts were found to differ between regions of the stem. Among these, 18 were novel isoforms of annotated genes and 187 were new genes (Data S10). For example, cellulose synthases (CESAs) *CESA1*,* CESA3* and *CESA6*, which are required for cellulose synthesis in primary cell walls in *Arabidopsis* (Desprez *et al*., 2007), were expressed predominantly in the apex (K6), apex‐IN2 (K3), IN1 (K4) and IN2 (K8). In the apex to IN2, where cells are undergoing cell expansion, expansins and cellulose synthases were highly expressed (Data S10). The high levels of expansins gene expression in IN1‐IN2 suggest that cell walls are loosening and cells are expanding during this developmental stage (Cosgrove, 2015). Although expansins were annotated in the first version of the *Populus* genome (Pt_v1), their expression patterns have not been described (Cosgrove, 2015; Sampedro *et al*., 2006). We identified two transcripts encoding *CSLD5* that are predominantly expressed in the apex and IN1‐2, although *CSLD5* was previously reported to play roles late in the cell cycle and to contribute to wall formation in new cells in the *Arabidopsis* shoot apex (Yang *et al*., 2016). The expression levels of *CESA9*,* CELLULOSE SYNTHASE‐LIKE A2* (*CSLA2*), *CSLA5* and *CSLA9* also peaked in the apex, IN1 or IN2.

Expression of *CESA4*,* CESA7* and *CESA8* (Taylor *et al*., 2003), which are involved in cellulose synthesis in the secondary cell wall in *Arabidopsis*, peaked in IN4‐5. We identified three new alternatively spliced isoforms encoding *CESA8*. Apart from cellulose synthase, two major alternatively spliced *COBRA‐like 4* (*COBL4*) transcripts were expressed predominantly in IN4‐5; *COBL4* has been shown to affect cellulose microfibril crystallinity in rice (Liu *et al*., 2013). It seems that the expression patterns of these genes are consistent with their known functions in secondary cell wall synthesis. But the functions of *CSLA09*,* GH9B3* and *BXL1* in secondary growth have not been reported previously and need to be studied in the future.

### Transcription factor dynamics during shoot development

**Figure 6:**
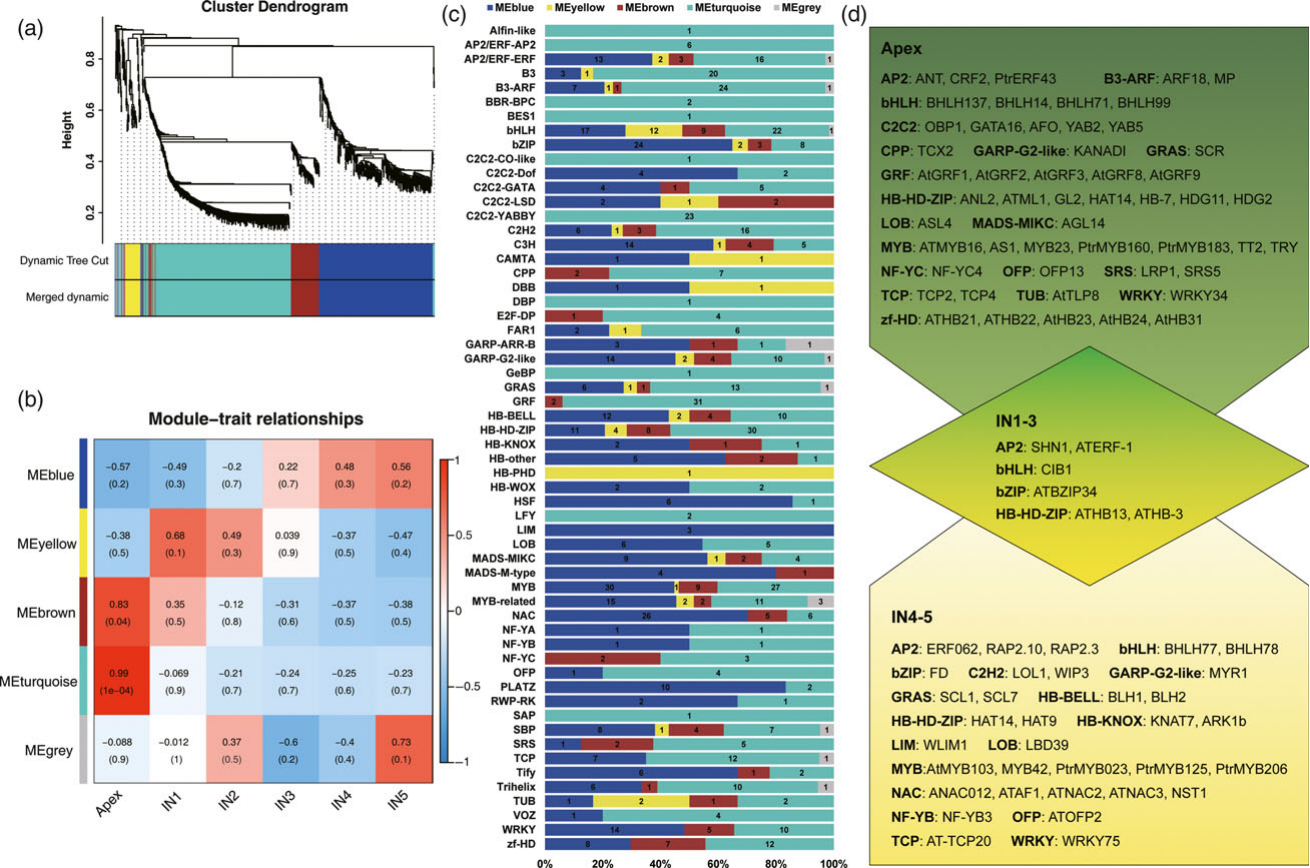
Weighted Gene Co‐expression Network Analysis (WGCNA) of transcription factors. (a) Cluster dendrogram of transcription factors based on expression levels in the six developmental zones (Apex, IN1, IN2, IN3, IN4 and IN5). Each branch represents a gene and each colour below represents a gene co‐expression module. Dynamic tree cut indicates the modules divided based on the gene clustering results. Merged dynamic indicates the modules divided by combining modules with similar expression patterns. (b) Heatmap of transcription factor expression patterns in different samples (Apex, IN1, IN2, IN3, IN4 and IN5). The expression patterns of five modules are shown by the heatmap. The colour bar indicates expression levels from low (blue) to high (red). (c) Distribution of transcription factor families in different WGCNA modules. Each colour represents a co‐expression module, and the numbers indicate the number of transcription factors in the module. (d) Transcription factors highly expressed in the apex, IN1‐3 and IN4‐5.

TFs play important roles in developmental transitions. In addition, many TFs that are important for shoot development in *Populus* have been identified (Dharmawardhana *et al*., 2010; Prassinos *et al*., 2005). Therefore, we investigated the expression patterns of TFs during the transition from primary to secondary growth. Of the 8347 TFs expressed during *Populus* shoot development, 1216 were differentially expressed along a developmental gradient (Figure 6a). To determine the co‐expression and correlation networks of all differentially expressed TFs, weighted correlation network analysis (WGCNA) was conducted. Five modules of highly correlated TFs that are highly expressed during different stages of *Populus* shoot development (coloured turquoise, blue, brown, yellow and grey in Figure 6b; Figure S4 and Data S11) were identified. Most TFs (403) belong to the turquoise module, in which peak expression of most TFs is in the apex. This suggests that TFs play an important role in stem cell maintenance and lateral meristem cell differentiation. TFs belonging to the brown (94) and blue (319) modules have decreased and increased expression from the apex to IN5, respectively. The yellow module contains the smallest number of TFs (58), which are most highly expressed in IN1‐IN2.

Using the *Arabidopsis* TF database TFDB 3.0 (Jin *et al*., 2014) and the TF prediction algorithm HMMER 3.0 (Finn *et al*., 2011), TFs were classified into 58 families. Family‐specific expression was observed in different regions of the shoot (Figure 6c). Expression of most AP2 (AP2‐ERF family), ARF (B3 family), YABBY (C2C2 family) and GRF genes peaked in the apex. Expression of most NAC, bZIP, PLATZ and HSF TFs peaked in IN4 and IN5. Expression of most ERF (AP2‐ERF family), G2‐like (GARP family), GRAS and BELL (HB family), LOB, MYB, MYB‐related, TCP, WRKY and zf‐HD TFs peaked in both the apex and IN4 or IN5 (Figure 6d).

Based on a previous report, MYB and NAC family TFs play important roles in secondary growth *in Arabidopsis* (Zhong *et al*., 2010). Consistent with this, in this study, different MYB family genes were expressed in the apex and IN4‐5. For example, *AS1/ATPHAN*, which maintains meristem activity and is required for growth and dorsoventrality of lateral organs in *Antirrhinum* (Waites *et al*., 1998) was predominantly expressed in the apex. But *PtrMYB206*, whose orthologue *MYB59* may be involved in cell division in *Arabidopsis* (Mu *et al*., 2009), was predominantly expressed during secondary growth. NAC family genes were also expressed predominantly in IN4‐5, including *NST1*,* SND1*,* ATAF1*,* ATNAC2* and *ATNAC3*. NSTs can directly activate *MYB46* and its close homologs (Zhao and Dixon, 2011), and *SND1* directly regulates *MYB46*, which is involved in secondary cell wall biosynthesis in *Arabidopsis* (Ko *et al*., 2012b; Zhong *et al*., 2007). *MYB46* orthologues are also expressed in IN4‐5 (Data S11).

Genes responsible for *Arabidopsis* shoot and root meristem development, such as *SCR*,* YABBY2*,* YABBY5*,* ASL4/LOB*, and so on, were expressed in the apex. In *Arabidopsis*,* SCR* is expressed in ground tissue in both the root and shoot during embryogenesis as well as postembryonically, and is required for distal determination of the root quiescent centre (Sabatini *et al*., 2003; Wysocka‐Diller *et al*., 2000). Other TFs, such as *YABBY2*,* YABBY5* and *ASL4/LOB*, have previously been shown to play roles in lateral organ initiation in meristems in *Arabidopsis* (Byrne *et al*., 2000; Rast and Simon, 2008; Sun *et al*., 2002), and the high expression levels of these genes in the apex suggests that they have similar functions in *Populus*.

Many hormone‐associated receptors that are also TFs, such as *CRF2*,* PtrERF43*,* ARF18* and *MP*, were expressed in the apex, including *SHN1* and *ATERF‐1* in IN1‐3, and *ERF062*,* RAP2.10* and *RAP2.3* in IN4‐5. Many of these genes, including *MP* and *ARF18*, have previously been found to play roles in embryo and procambium development in *Arabidopsis* (Miyashima *et al*., 2013; Weigel and Jurgens, 2002). And new isoforms of *MP* (PB9131.3) and *ARF18* (PB.19400.8) were identified in this study. In contrast to previous studies in *Arabidopsis*, which showed that *CRF2* is induced in response to cytokinin in whole seedlings and is important for root apical meristem development and lateral root initiation after germination (Jeon *et al*., 2016; Raines *et al*., 2016), we found that *CRF2* was predominantly expressed in the SAM in *Populus*. The expression of *WAX INDUCER1 (WIN1)/SHN1* in the apex and the expression of *long‐chain acyl‐CoA synthetase* (*LACS2*) in the apex and IN1 is consistent with a previous finding that *WIN1*/*SHN1* modulates cuticle permeability in *Arabidopsis* by regulating genes encoding cutin pathway enzymes, such as *LACS2* (Kannangara *et al*., 2007). Real‐time PCR analysis of *AFO*,* MP*,* SHN1* and *ARK1b* expression indicated that *AFO* and *MP* were expressed predominantly in the apex, while *SHN1* and *ARK1b* were expressed predominantly in IN1‐2 and IN4‐5, respectively (Figure S5). We observed *ARK1b* expression in IN4‐5; this gene was previously reported to regulate the SAM, vascular cambium, and wood formation in *Populus* (Groover *et al*., 2006; Liu *et al*., 2015).

## Conclusions

In this study, we used Pac‐Bio Iso‐Seq and RNA‐Seq to profile changes in gene expression during the transition to primary and secondary growth in *Populus*. We identified 87 150 transcripts, including new alternatively spliced isoforms of previously annotated genes as well as novel genetic loci, and used this information to update the *Populus* annotation. GO enrichment analysis of genes differentially expressed during *Populus* stem development revealed that although similar processes, such as cell division and oxidation‐reduction, are important during both primary and secondary growth, different genes participate in these processes in different stages of development. We also found that different types of TFs tend to be expressed at different stages. For example, AP2, ARF, YABBY and GRF TFs are highly expressed in the apex, whereas NAC, bZIP, PLATZ and HSF TFs are likely to be important for the transition to secondary growth. There are many transcripts expressed predominantly in primary and secondary growth zone according to our study, but their functions are still unknown. These genes are a potential resource to study primary and secondary growth, especially the key transition from primary to secondary growth.

## Materials and methods

### Sample collection and anatomic analysis

*Populus deltoides × P. euramericana cv* ‘*Nanlin895*’ was grown in a phytotron with a light and dark cycle of 16 and 8 h at 23 °C. Apical and stem samples were collected from apex, the first internode (IN1), the second internode (IN2), the third internode (IN3), the fourth internode (IN4) and the fifth internode (IN5) from plants as shown in Figure 1. Each sample was pooled from 15 plants, and 3 biological repeats were performed for a total of 45 plants for each region of the stem. Three mixed samples from 45 plants were collected for PacBio full‐length sequencing.

Stem samples were cut by knife blade, fixed in FAA, and then dehydrated through a gradient of ethanol and embedded in LR White resin (London Resin Company Ltd). One‐micrometre‐thick sections were cut with a Leica EM UC7 microtome (Leica Microsystems IR GmbH, Wetzlar, Germany), stained with toluidine blue O (Sigma‐Aldrich, St. Louis), and viewed with an optical microscope.

### RNA quantification and assessment of quality

RNA degradation and contamination were assessed on 1% agarose gels. RNA purity was checked using a NanoPhotometer spectrophotometer (IMPLEN, CA). RNA concentration was measured using the Qubit RNA Assay Kit and a Qubit 2.0 Fluorometer (Life Technologies, CA). RNA integrity was assessed using the RNA Nano 6000 Assay Kit and the Agilent Bioanalyzer 2100 system (Agilent Technologies, CA).

### Illumina transcriptome library preparation and sequencing

A total of 1 μg RNA per sample was used as input material to generate sequencing libraries using the NEBNext UltraTM RNA Library Prep Kit for Illumina (NEB) following the manufacturer's recommendations, and index codes were added to attribute sequences to a specific sample. Briefly, mRNA was purified from total RNA using poly‐T oligo‐attached magnetic beads. Fragmentation was carried out using divalent cations under elevated temperature in the NEBNext First Strand Synthesis Reaction Buffer (5×). First strand cDNA was synthesized using random hexamer primers and M‐MuLV Reverse Transcriptase (RNase H‐). Second strand cDNA synthesis was subsequently performed using DNA polymerase I and RNase H. Remaining overhangs were converted into blunt ends via exonuclease/polymerase activities. After adenylation of 3′ ends of DNA fragments, NEBNext Adaptors with hairpin loop structures were ligated to prepare for hybridization. In order to select cDNA fragments 200–250 bp in length, the library fragments were purified with the AMPure XP system (Beckman Coulter, Beverly). Then 3 μL USER Enzyme (NEB) was incubated with size‐selected, adaptor‐ligated cDNA at 37 °C for 15 min followed by 5 min at 95 °C. Then PCR was performed with Phusion High‐Fidelity DNA polymerase, Universal PCR primers and Index (X) Primer. Finally, PCR products were purified (AMPure XP system) and library quality was assessed on the Agilent Bioanalyzer 2100 system. The clustering of index‐coded samples was performed on a cBot Cluster Generation System using the TruSeq PE Cluster Kit v4‐cBot‐HS (Illumina) according to the manufacturer's instructions. After cluster generation, the libraries were sequenced on an Illumina Hiseq X Ten platform, and paired‐end reads were generated.

### PacBio Iso‐Seq library preparation and sequencing

The sequencing library was prepared according to the Iso‐Seq protocol as described by Pacific Biosciences (P/N100‐377‐100‐05 and P/N100‐377‐100‐04). The SMARTer PCR cDNA Synthesis Kit was used to synthesize cDNA from the same RNA samples used for Illumina sequencing. After 23 cycles of PCR amplification, products were size selected using the BluePippin Size Selection System with the following bins for each sample: 1–2, 2–3 and 3–10 kb. The amplified cDNA products were used to generate SMRTbell Template libraries according to the Iso‐Seq protocol. Libraries were prepared for sequencing by annealing a sequencing primer and adding polymerase to the primer‐annealed template. The polymerase‐bound template was bound to MagBeads and sequencing was performed on a PacBio RSII instrument.

### Illumina data analysis

Raw data (raw reads) in fastq format were first processed using in‐house perl scripts. In this step, clean data (clean reads) were obtained by removing reads containing adapters, reads containing poly‐N and low‐quality reads. These clean reads were then mapped to the reference genome sequence using Tophat2. Only reads with a perfect match or one mismatch were further analyzed and annotated based on the reference genome. Gene expression levels were estimated by FPKM. Differential expression analysis between two conditions/groups was performed using the DESeq R package (1.10.1) (Anders, 2010). The resulting *P* values were adjusted using the Benjamini and Hochberg's approach for controlling the false discovery rate (Anders, 2010). Genes identified by DESeq with FDR ≤0.01 and FC ≥2 were defined as differentially expressed. K‐means clustering was conducted based on Pearson correlation of gene expression profiles (Walvoort *et al*., 2010).

### PacBio data analysis

SMRT‐Analysis software package v3.0 (https://github.com/ben-lerch/IsoSeq-3.0/blob/master/README.md) was used for Iso‐Seq data analysis. First, ROIs were generated using the minimum filtering requirement of 0 or greater passes of the insert and a minimum read quality of 75. Then, FLNC (full‐length non‐chimeric) reads containing the 5′ and 3′ adapters used in the library preparation as well as the poly (A) tail were identified.

### lncRNA identification from PacBio sequences

Transcripts were screened for putative lncRNAs using four computational approaches, including coding‐non‐coding index (CNCI), coding potential calculator (CPC), coding potential assessment tool (CPAT), and Pfam database to identify non‐protein coding RNA candidates and putative protein‐coding RNAs from the unknown transcripts. Putative protein‐coding RNAs were filtered out using a minimum exon length and number threshold. Transcripts with lengths greater than 200 nt and with more than two exons were selected as lncRNA candidates. This threshold was chosen in order to filter abundant low‐expression, low‐confidence and single‐exon transcripts (Bogu *et al*., 2016; Peng *et al*., 2018; Yang *et al*., 2017). The putative lncRNAs were further screened using CPC, CNCI, CPAT and Pfam, which have the power to distinguish protein‐coding genes from non‐coding genes.

### Fusion transcript identification from PacBio sequences

The following criteria were used to define fusion transcripts: (a) full‐length transcripts map to two or more loci in the genome; (b) each mapped locus must align with at least 5% of the transcript; (c) the combined alignment coverage must be at least 95%; and (d) the mapped loci must be at least 10 kb apart. Furthermore, Illumina short reads generated from the HiSeqX Ten platform were used to validate candidate fusion transcripts. After mapping using bowtie2, junction‐spanning reads and discordant read pairs were found to support the candidate fusion transcripts.

### Functional annotation of transcripts

Transcripts were annotated by conducting blastx searches against public databases, including the NCBI non‐redundant protein database (Nr), NCBI non‐redundant nucleotide database (Nt), Swiss‐Prot, Protein Family (Pfam), Gene Ontology (GO) and the Kyoto Encyclopedia of Genes and Genomes (KEGG), with an *E*‐value threshold of 10^−5^.

### *Arabidopsis* homologous genes searches

Transcripts were used as queries in translated nucleotide BLAST (BLASTX) searches against *Arabidopsis* protein sequences in the *Arabidopsis* Information Resource 10 database to obtain the closest *Arabidopsis* homolog (*E*‐value ≤e−10).

### GO enrichment analysis

GO enrichment analysis was performed using topGO (Alexa *et al*., 2006) with Fisher's exact test, and the negative log10 transformed *P* values were visualized using heatmaps as previously described (Xue *et al*., 2013).

### Transcription factor identification and analysis

The set of *Arabidopsis* TFs in Plant TFDB 3.0 (Jin *et al*., 2014) was used as the reference TF database. The Transcription Factor Prediction algorithm HMMER 3.0 (Finn *et al*., 2011) was used to identify TFs and assign genes to different families. The best BLAST hits had maximal *E*‐values of 1e−10. The WGCNA (v1.42) package (Langfelder and Horvath, 2008) in R was used to construct co‐expression networks. Genes with FPKM values >1 were used for WGCNA co‐expression network analysis. The modules were obtained using the automatic network construction function blockwiseModules with default settings.

### Isoform identification and quantitative real‐time polymerase chain reaction (qRT‐PCR)

Primers were designed from specific PacBio‐seq isoform sequences (Table S1). Isoform‐specific primers were designed to distinguish between different alternative splicing events. Reverse transcription of 3 μg mRNA was used done using the M‐MLV first strand kit (ThermoFisher Scientific, MA). QRT‐PCR reactions were performed using SYBR Premix Ex Taq (Takara, Shiga, Japan) on a LightCycler 480 system (Roche, Basel, Switzerland). The amplification program consisted of 30 s of initial denaturation at 95 °C, followed by 30 cycles of 10 s at 95 °C, 20 s at 60 °C and 20 s at 72 °C, and ended with a final extension step at 72 °C for 20 s. All samples were normalized to the reference gene tubulin from Populus to analyze the isoform expression level. The final relative expression level was calculated using the formula *F* = 2^−ΔΔCt^.

## Conflict of interest

The authors declare no conflict of interest.

## Supporting information


**Figure S1** Schematic of the computational and bioinformatics pipeline for joint analysis of PacBio Iso‐Seq and RNA‐seq reads.
**Figure S2** RT‐PCR verification of alternative splicing events for three genes.
**Figure S3** Analysis of expression correlation between samples.
**Figure S4** Heatmaps of different transcription factor WGCNA modules.
**Figure S5** Expression verification of isoforms in different sections of the *Populus* stem.
**Table S1** Sequences of primers used for alternative splicing and gene expression verification

**Data S1** PacBio Iso‐Seq output analysis

**Data S2** Transcripts from new genes

**Data S3** Alternative splicing events

**Data S4** Long non‐coding RNAs

**Data S5** Fusion transcripts

**Data S6** Mapping ratios of RNA‐seq sequences

**Data S7** Differently expressed transcripts

**Data S8** K‐means clusters

**Data S9** GO enrichment analysis

**Data S10** Cell wall‐associated transcripts

**Data S11** Transcription factors
