# Supplementary material for: The developmental dynamics of the Populus stem transcriptome
Source: Plant Biotechnol J. 2018 Jun 25;17(1):206–19. doi: 10.1111/pbi.12958 (PMC6330540; doi:10.1111/pbi.12958)
Supplement: Supplementary file 1 — Figure S1 Schematic of the computational and bioinformatics pipeline for joint analysis of PacBio Iso‐Seq and RNA‐seq reads. Figure S2 RT‐PCR verification of alternative splicing events for three genes. Figure S3 Analysis of expression correlation between samples. Figure S4 Heatmaps of different transcription factor WGCNA modules. Figure S5 Expression verification of isoforms in different sections of the Populus stem. Table S1 Sequences of primers used for alternative splicing and gene expression verification [file PBI-17-206-s010.pdf]

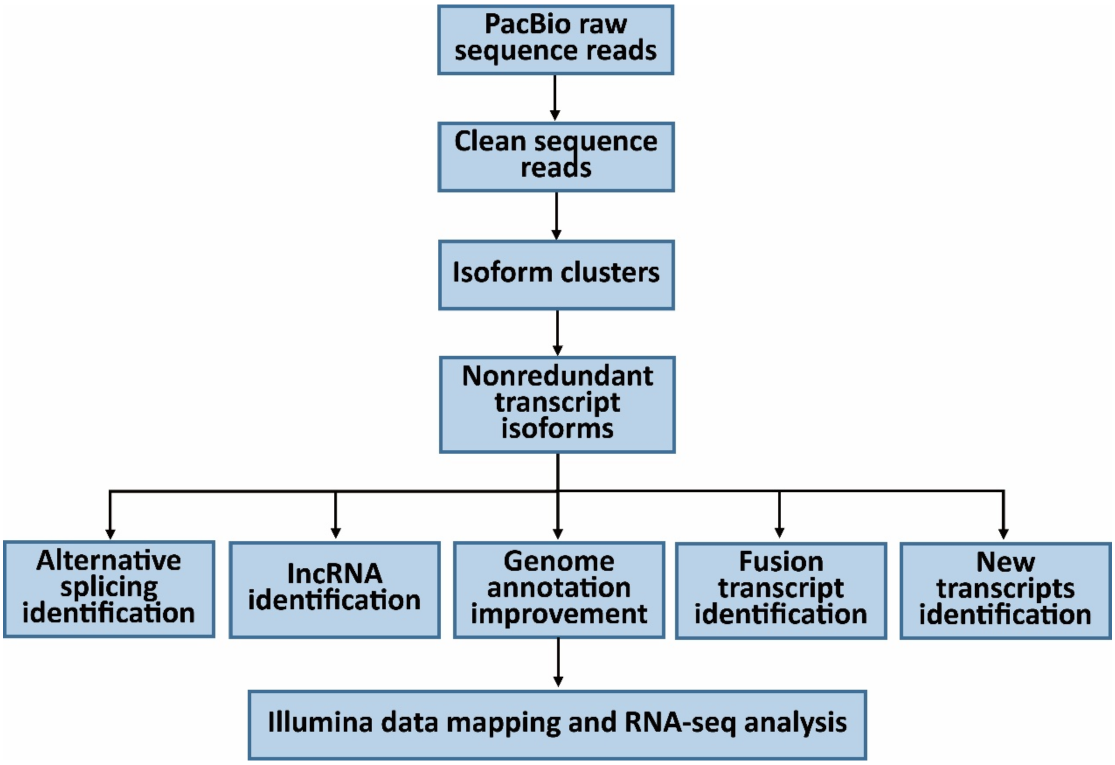

Figure S1 Schematic of the computational and bioinformatics pipeline for joint analysis of PacBio Iso-Seq and RNA-seq reads.

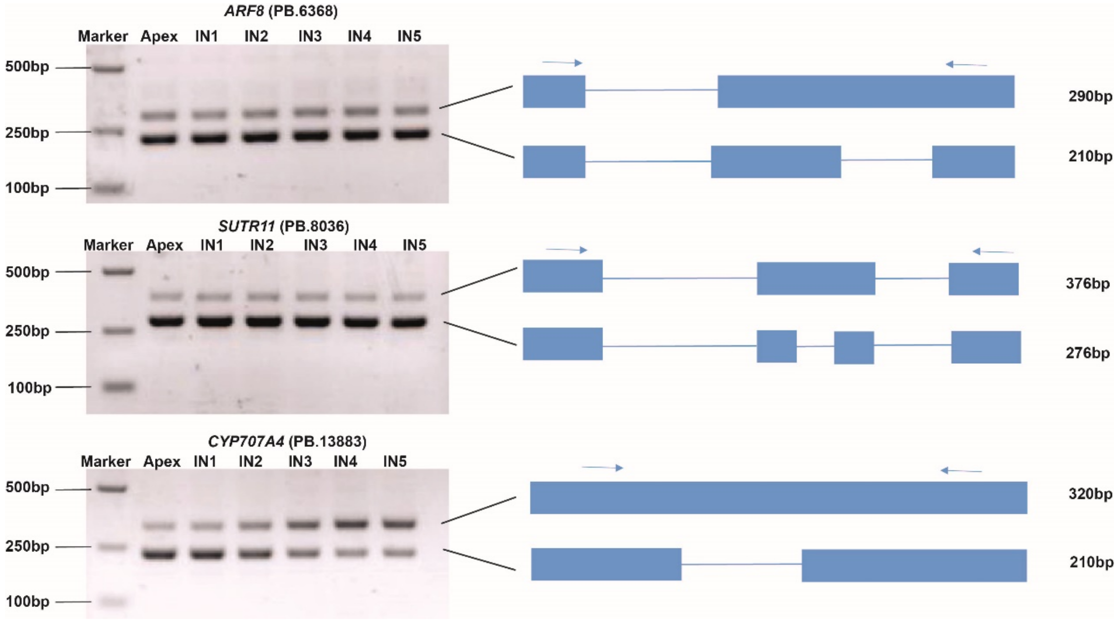

Figure S2 RT-PCR verification of alternative splicing events for three genes.

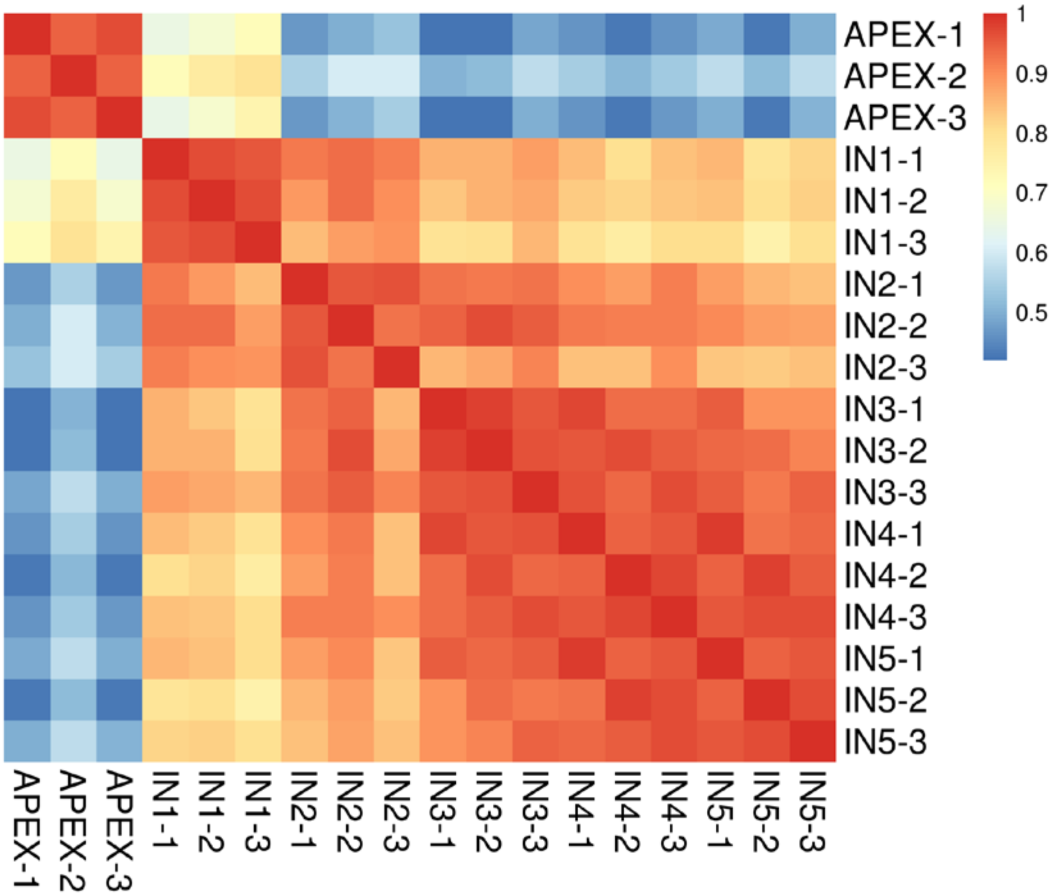

Figure S3 Analysis of expression correlation between samples.

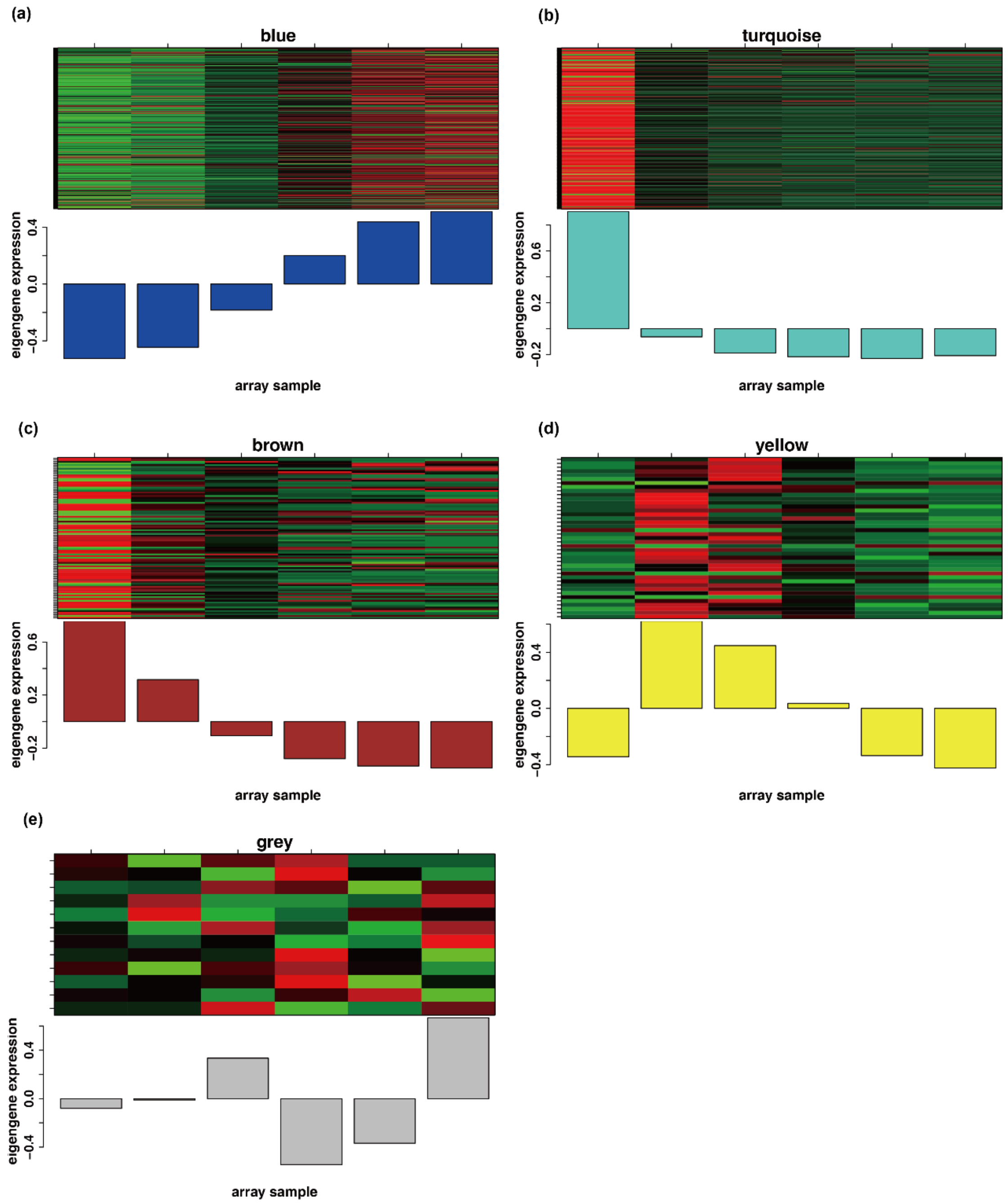

Figure S4 Heatmaps of different transcription factor WGCNA modules.

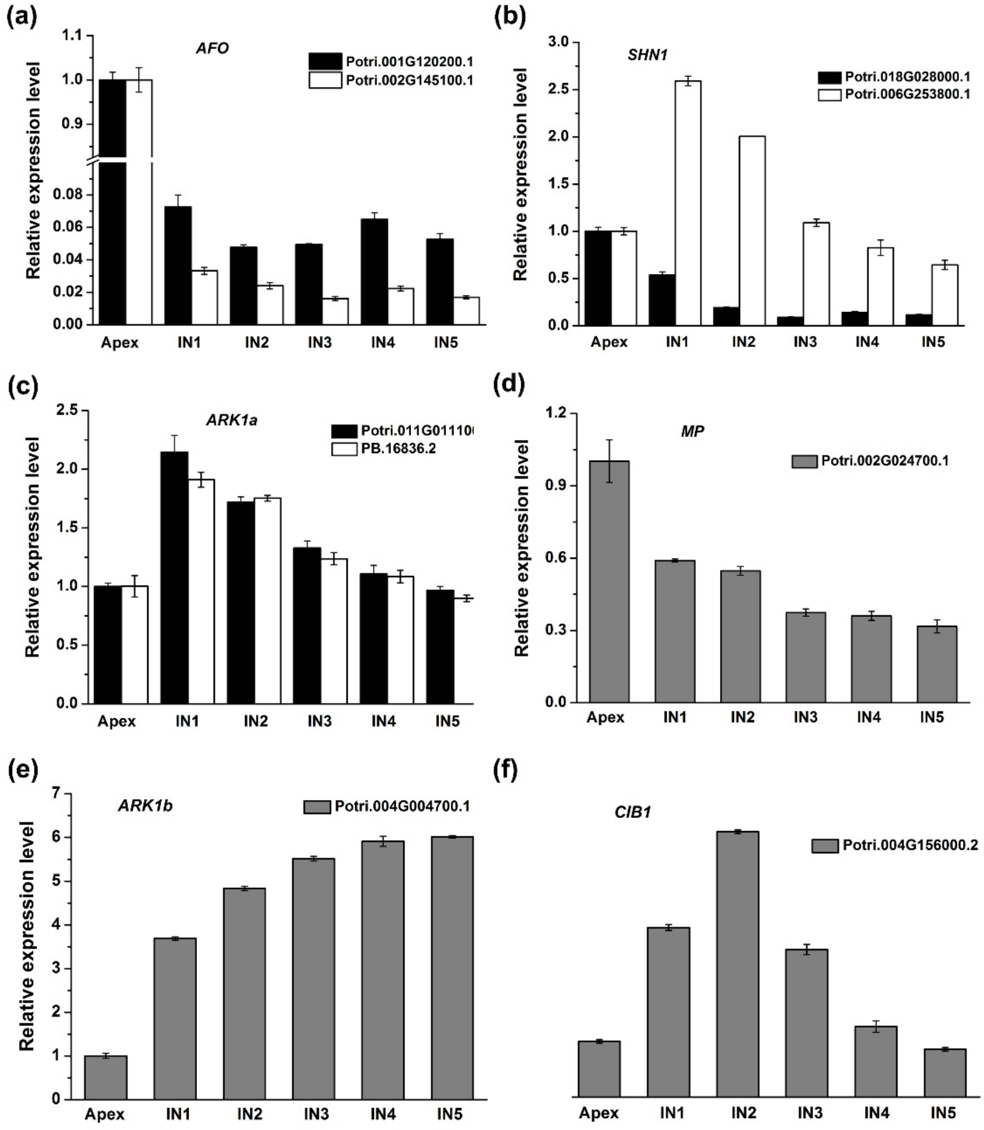

Figure S5 Expression verification of isoforms in different sections of the Populus stem.

(a) Expression of two major transcripts encoding AFO in different stem.

(b) Expression of two major transcripts encoding SHN1 in different stem.

(c) Expression of two major transcripts encoding ARK1a in different stem.

(d) Expression of major transcripts encoding MP in different stem.

(e) Expression of major transcripts encoding ARK1b in different stem.

(f) Expression of major transcripts encoding AFO in different stem.

Table S1 Sequences of primers used for alternative splicing and gene expression validation.

Gene name	Primer	Sequence (5'-3')
AFO	1202.1L	TTCACTTCGGTCTCATGCCC
	1202.1R	CGTAGCAGCTCCACTTCTTAG
AFO	1451.1L	TACCCACTGGTCAGACAATCC
	1451.1R	CAAAGCTGCTCGGAGGAAGA
MP	247.1L	AAGTTGGAGTGCAGGCTAATAG
	247.1R	TGTGGTGGTGGTCTTGTAGC
SHN1	2538.1L	GACACCGGACCTTCCTCGAC
	2538.1R	GATTGGCAACTGTTTATCTGTG
SHN1	280.1L	TGAGATTGAGCCGGAAAATG
	280.1R	TGTCTAAACCCCATAAGAAATAG
ARK1a	16836.2L	ATTGATAACCGTGCCCCATC
	16836.2R	TGAACGATCAAATCGAGGAA
ARK1a	111.1L	ATGCTGTTCTCAAGTGCTGTT
	111.1R	TGGCTCCAAAACAGGCTTAA
ARK1b	47.1L	ACTGTGAGGGAGGGAGGGAG
	47.1R	AGAAACCAAGCAGGAAAACG
Tublin	TUBLIN-L	CTTGATCGAGTGAGGAAGTTGGCTG
	TUBLIN-R	ACGACTGCCGTTGAAACCTGAGGAG
ARF8	PB.6368L	CATTACAGCCTTTGACACCG
	PB.6368R	TGGAGATCCCTGGCGATAAG
SUTR11	PB.8036L	AGGTTCCTGGTGTTTTGATT
	PB.8036R	TCAGAGGCGTGGAGCTTATC
CYP707A4	PB.13883L	AATGGGCTGGCCTTTTGTAG
	PB.13883R	GCTGAAGGGCCAATCATCTT
